# MicroRNA-induced negative regulation of TLR-5 in grass carp, *Ctenopharyngodon idella*

**DOI:** 10.1038/srep18595

**Published:** 2016-01-04

**Authors:** Xiao-Yan Xu, Yu-Bang Shen, Jian-Jun Fu, Hong-Yan Yu, Wen-Ji Huang, Li-Qun Lu, Jia-Le Li

**Affiliations:** 1 Key Laboratory of Exploration and Utilization of Aquatic Genetic Resources, Shanghai Ocean University, Ministry of Education, Shanghai 201306, China; 2 E-Institute of Shanghai Universities, Shanghai Ocean University, 999 Huchenghuan Road, Shanghai 201306, PR China; 3 National Pathogen Collection Center for Aquatic Animals, College of Fisheries and Life Science, Shanghai Ocean University, 999 Huchenghuan Road, Shanghai 201306, PR China

## Abstract

MicroRNAs (miRNAs) are endogenous small non-coding RNAs that play crucial roles in numerous biological processes. However, the role of miRNAs in antibacterial defence in fish has not been fully determined. Here, we identified that nine miRNAs are differentially expressed in kidney between susceptible and resistant grass carp strains. Analysis of spatial and temporal miRNA expression patterns suggests that cid-miRn-115 and miR-142a-3p are potential regulators of anti-bacterial activity. Overexpressing of cid-miRn-115 and miR-142a-3p results in a visible change in *Ctenopharyngodon idella* kidney (CIK) cells immune effector activity. Bioinformatics analysis and overexpressing assay shows that cid-miRn-115 and miR-142a-3p directly regulate *tlr5* expression. cid-miRn-115 and miR-142a-3p overexpressing leads to a significant decrease in *tlr5* expression in CIK, thereby repressing its downstream genes, such as *il-1β*, *il-8* and *tnf-α*. These findings provide a novel insight into the determination of anti-bacterial compounds in grass carp.

MicroRNAs (miRNAs) are a class of genome-encoded small RNAs that post-transcriptionally regulate the expression of cellular mRNAs exhibiting partial or full complementary miRNA-binding sites^1^. Several studies have revealed that miRNAs play important roles in the control of many biological processes, such as cell differentiation, proliferation and apoptosis, development, immunity, metabolism and stem cell maintenance^2^. It is now evident that aberrant miRNA expression in the immune system is sufficient to cause disease, thus indicating that proper regulation of miRNA expression is crucial for disease prevention^3^. MiRNAs have well-established roles in eukaryotic host responses to viruses, and to extracellular and invasive bacterial pathogens^4^. The involvement of miRNAs in bacterial infections was first discovered in plants; *Arabidopsis* miR-393 was shown to contribute to resistance against the extracellular pathogen *Pseudomonas syringae*, presumably by repressing auxin signalling^5^. Based on studies of plant immunity and defence mechanisms, it has been suggested that eukaryotes restrict bacterial pathogens by a balanced induction of miRNAs that repress negative defence regulators as well as suppressing miRNAs that repress positive effectors of defence^6^. Therefore, the study of miRNA-mediated host-bacteria interactions may further elucidate the mechanisms underlying bacterial infection and host counteraction.

The grass carp (*Ctenopharyngodon idella*) is an economically important cultured fish species in China; its farming results in the largest yield for a single species worldwide^7^. In recent years, the rapid development of the grass carp aquaculture industry is accompanied with increasingly severe infections, caused by viruses, bacteria and parasites, and resulting in great economic losses^8^. Investigating mechanisms of host immunity will greatly improve the countrol and prevention of disease. Recently, numerous immune-related genes have been successfully identified in grass carp using various molecular methods. Following the successful completion of furture aquatic animal genome projects, studies of the regulation mechanisms of noncoding RNAs responding to these genes will become increasingly important.

*Aeromonas hydrophila,* which was first recognized as the causal agent of haemorrhagic septicaemia^9^, is considered to be the dominant cause of motile aeromonad septivaemia (MAS) in China^10^, and appears to be distributed globally. *A. hydrophila* is present in a wide variety of foods (introduced from water, animal faces or food handlers), and, thereby, has the potential to be a significant food-borne pathogen, representing a serious public health concern^11^. Regardless of the important regulatory role of miRNAs in the host immune system, no studies have been conducted on miRNA transcriptomes and their expression profiles related to immune response to foreign challenge in *A. hydrophila*. At present, the pathogenic mechanism of *A. hydrophila* infection is poorly understood and the involvement of miRNAs during *A. hydrophila* infection has not been reported. We thus aimed to determine the repertoire of miRNAs expressed in the kidney of grass carp and to use this repertoire to study the responses of this teleost to *A. hydrophila* infection. Solexa sequencing technology was used in this study to sequence and analyse miRNA libraries generated from susceptible grass carp (SGC) and resistant grass carp (RGC) strains.

In this study, we aimed first to characterize the expression of miRNA in the grass carp in relation to MAS, and second to evaluate the diagnostic potential of the investigated miRNAs as biomarker for MAS. Using two kidney microRNA transcriptomes from SGC or RGC infected with a highly pathogenic *A. hydrophila*, we show that the kidney exhibit different miRNA expression levels. Additionally, we show that these miRNAs are differentially expressed in the immune related tissue and clear time-dependent expression pattern after the bacterial challenge, suggesting their potential use as biomarkers for MAS conditions.

## Materials and Methods

### Animals used

Grass carp with an average weight of 50 g were cultured individually in Wujiang National Farm of Chinese Four Family Carps, Jiangsu Province, China. Animals were raised at 28 °C in 400 L aerated tanks for one week before the experiment and fed twice daily (in the morning and late in the afternoon) at a ratio of 5% of the total biomass. All experiments were conducted under the guidance of the Care and Use of Laboratory Animals in China. This research was approved by the Committee on the Ethics of Animal Experiments of Shanghai Ocean University, China.

Grass carp were divided into three groups (30 animals per group) for the injection experiments. The conditions were identical among the tanks and the fish were randomly distributed into different tanks. Two groups were maintained in two aquariums and intraperitoneally injected with *A. hydrophila* AH10 (Aquatic Pathogen Collection Centre of Ministry of Agriculture, China) at a dose of 7.0 × 10^6^ cells suspended in 100 μl PBS per fish. The third group was injected with PBS as control. All fish were observed every 4 h for any mortality and samples were collected until the termination of the experiment at 240 h post-challenge. Grass carp that died in the first 72 h post-challenge were classified as susceptible grass carp (SGC), whereas the animals that survived over 240 h post-challenge were considered resistant grass carp (RGC). The kidney tissues of randomly-selected three fish from both the susceptible and resistant groups were collected and, labeled as SGC and RGC, respectively. Approximately 0.5 g kidney tissue was cut and kept at −80 °C until RNA isolation. Total RNA was extracted using TRIzol reagent (Invitrogen, Carlsbad, CA, USA) and stored at −80 °C. RNA was quantified using a NanoDrop Spectrophotometer 2000c (NanoDrop Technologies, Wilmington, DE, USA), and its quality was assessed on a 2100 Bioanalyzer (Agilent Technologies, Palo Alto, CA, USA). All samples used had λ_260/280_ and λ_260/230_ ratios > 1.8.

### Small RNA library construction and Illumina sequencing

RNA samples were harvested from kidney of SGC and RGC animals, and immediately frozen in liquid nitrogen. Small RNA libraries were constructed using a TruSeq Small RNA Sample Prep Kits (Illumina, San Diego, Califomia, USA). Approximately 20 μg of small RNA was submitted for sequencing. Briefly, the Solexa sequencing was performed as follows: RNA was purified by polyacrylamide gel electrophoresis (PAGE) to enrich for the molecules in the range of 17–27 nucleotides, and was then ligated with 5′ and 3′ adapters. The resulting samples were used as templates for cDNA synthesis, followed by PCR amplification. The obtained sequencing libraries were subjected to Solexa sequencing-by-synthesis method. After the run, image analysis, sequencing quality evaluation and data production summarization were performed with Illumina/Solexa pipeline.

### Basic analysis of sequencing data

The small RNA sequence reads were pre-processed, excluding low-quality reads (ambiguous N and length <18 nt) as well as 3′ adapter, 5′ adapter and poly(A) sequences. The resulting clean reads were aligned against Rfam, allowing a maximum mismatch of 2 nt to remove noncoding RNA, such as rRNA, tRNA, snRNA, and snoRNA. Obtained sequences were then compared with grass carp transcriptome^8^ to classify mRNA degradation. The remaining sequences were analyzed by BLAST search against Sanger miRBase (version 19.0). Sequences in our libraries that were identical or related (four or fewer nucleotide substitutions) to sequences from grass carp were identified as conserved miRNAs. Reads that did not match any database above were marked as cid-miRn. The secondary structures of the predicted miRNAs^12^ were confirmed by RNAfold (http://rna.tbi.univie.ac.at/cgi-bin/RNAfold.cgi).

### Differentially expressed genes between the SGC/RGC libraries

Gene expression levels were calculated using the transcripts per million clean tags (TPM) method^13^. The calculation of unigene expression levels and the identification of unigenes that were differentially expressed between the libraries were performed by DEGseq^14^ based on TMM normalized counts. The settings “q.value < 0.01^15^” and “|log2.Fold change.normalized| > 1” were used as thresholds for judging significant differences in transcript expression.

### Real-time RT-PCR analyses of miRNAs

Total RNA was extracted using TRIzol reagent (Invitrogen) according to the manufacturer’s instruction. RNA integrity was assessed by electrophoresis on 1.0% agarose gel. Three grass carp from each groups were included in qRT-PCR For mRNA quantification, reverse transcription was performed using a High Fidelity primeScript RT-PCR Kit as instructed (Takara, Dalian, China). MiRNA abundance was detected using stem-loop PCR method, and miR-192 expression was detected as internal control^16^. All reactions were performed in triplicate on the CFX96 Real-time PCR Detection System (Bio-Rad, Hercules, CA, USA). The relative gene or miRNA expression was detected using the comparative threshold cycle (CT) method also referred to as the 2^−ΔΔCt^ method. One-way ANOVA tests were performed using SPSS 20 to determine significant differences.

### Effect of pEGFP-N1-CiTLR5, cid-miRn-115 and miR-142a-3p on invasion of A. hydrophila in CIK cells

The ORF of *Ctenopharyngodon idella tlr5* (*Citlr5*) was amplified from grass carp cDNA and individually cloned into the pEGFP-N1 vector (Promega) by directional cloning. Invasion and proliferation assays were performed as previously described^17^. Using the Lipofectamin 2000 (Invitrogen) transfection reagent, 2 × 10^5^ *Ctenopharyngodon idella* kidney (CIK) cells cellswere transfected with 1 μg pEGFP-N1-CiTLR5 plasmids or 50 nM cid-miRn-115 and miR-142a-3p agomir (GenePharm, China). Twenty-four hours later, the cells were infected with *A. hydrophila* a the dose of 2.0 × 10^3^ cells for 45 min at 28 °C. The cells were then gently washed by PBS, and fresh M199 containing 100 μg ml^−1^ gentamicin was supplemented to kill extracellular bacteria for 1 h. After 15 min incubation with 1% Triton X-100, the cell lysates obtainedwere plated in serial dilutions to assess the number of bacteria that successfully entered CIK cells. The experiment was performed in triplicate. The statistical difference was calculated using Student’s t-test and was considered significant at *P* < 0.05

### Induction of pEGFP-N1-CiTLR5, cid-miRn-115 and miR-142a-3p on the expression of three immune factors *in vitro*

Four micrograms of pEGFP-N1-CiTLR5, cid-miRn-115 and miR-142a-3p was transfected into 1 × 10^6^ CIK cells. After 24 h, total RNA was extracted and reverse transcribed, as described above. Using the sequences from transcriptome^8^ as templates (*Ciil-1β*, *Ciil-8* and *Citnf-α*), we designed primers (Table 1) for quantitative reverse transcription PCR (qRT-PCR) to test the gene expressions levels of these immune factors following CiTLR5, cid-miRn-115 and miR-142a-3p overexpression. The relative expression levels of the genes were normalized to the expression of *18s rRNA*. Data were calculated by the 2^−ΔΔCT^ method. The assay was performed in triplicate.

## Results

### Overview of the high-throughput sequencing data

**Figure 1:**
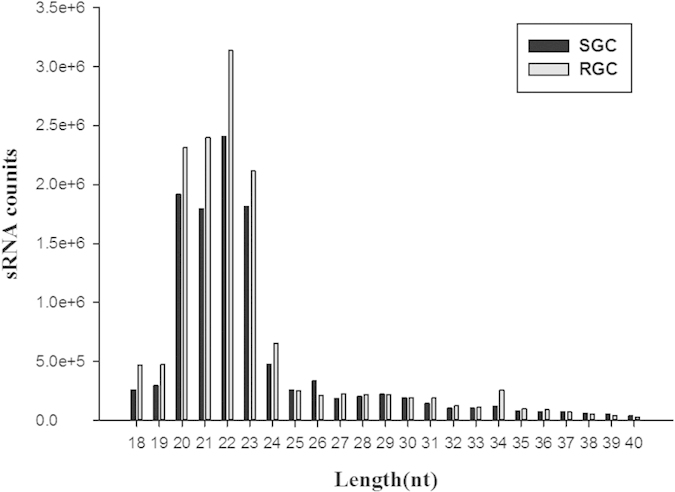
Length distribution and reads of small RNAs from *A. hydrophila*- susceptible (SGC) and-resistant grass carp (RGC) libraries.

Two sRNA libraries derived from pooled kidney tissues obtained under SGC and RGC, were constructed and sequenced using the Illumina deep-sequencing technology. In total, 13,284,378 and 16,095,116 raw reads were acquired from the SGC and RGC libraries, respectively. After excluding the low-quality tags, adapter sequences, polyA/T/G/C sequences and sequences shorter than 18 nt and longer than 40 nt, 11,218,000 (84.45% of the raw reads) and 13,941,337 (86.62% of the raw reads) clean reads were obtained, respectively (Table 2). The length distribution of the clean reads was analysed (Fig. 1). The majority of the small RNA (sRNA) sequences ranged from 20 to 23 nt in both libraries. The sRNA library under SGC and RGC showed a peak distribution at 22 nt. This supports the notion that mature miRNAs are evolutionary conserved, small, ∼22 nt, noncoding RNAs^18^.

### Discovery of miRNAs in grass carp

**Figure 2:**
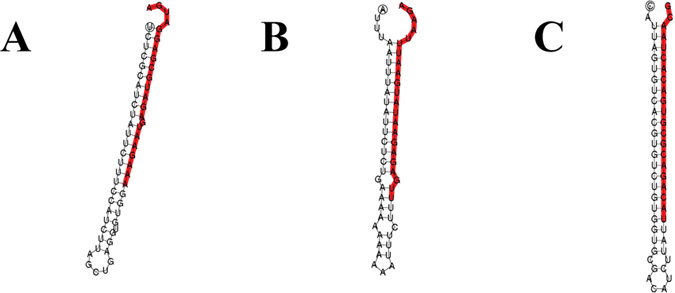
Predicted hairpin structures of grass carp coding candidate miRNAs. Dominant forms of the mature miRNAs are indicated in red. A: cid-miRn-100, B: cid-miRn-101, C: cid-miRn-102.

After mapping to the grass carp transcriptome, a total of 3,301,265/4,905,728 small RNA were mapped. Among them, 61 miRNAs were known in miRBase 19, and the remaining 116 miRNAs were not found to possess homology to any known metazoan miRNAs, suggesting a possible species-specificity, and were labelled cid-miRn. The miRNA could be distinguished from other sRNAs through a characteristic hairpin structure fold by the flanking sequences. The precursor sequences of the 116 putative candidate miRNAs could form the canonical hairpin structure, and the secondary structure for some candidates (the cid-miRn-100, cid-miRn -101 and cid-miRn-102) were are in Fig. 2.

### MiRNA expression profiles

High-throughput sequencing technology is not only an alternative means to identify small RNAs but also an useful tool to assess their expression profiles, in which the number of reads can serve as an index for the relative abundance of diverse miRNAs^19^. In the present study, the transcripts per million (TPM) of these miRNA sequences ranged from 0.44 to 155,651.08, indicating great variation in the expression level. The average number of TPMs for novel miRNAs was lower than those of conserved miRNAs (16.04 versus 9,511.31), indicating that the novel miRNAs are usually weakly expressed, whereas conserved miRNAs are highly expressed (see Table S1 and S2 for further details), consistent with the phenomenon observed in other fish species^20^. Among these identified miRNAs, the most abundant miRNA was miR-101a, with a total TPM value of 308,750.21 in SGC and RGC libraries, followed by miR-146b (TPM value 168,822.24) and miR-126a-3p (TPM value 139,223.22).

**Figure 3:**
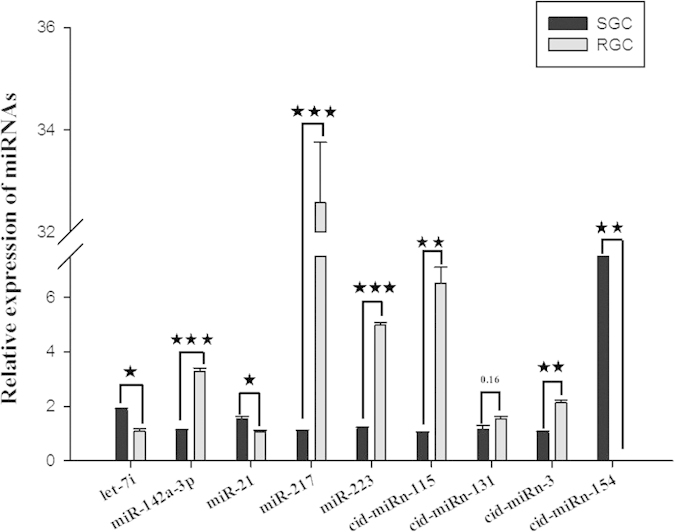
qPCR of conserved miRNAs in the *A. hydrophila*-susceptible (SGC) and -resistant grass carp (RGC) libraries. The expression of selected miRNAs in SGC or RGC was validated by qPCR amplification, using a specific primer for each miRNA. The fold change in expression was calculated based on the level of miR-192 expression (used as a control for the normalization). The data were obtained from three independent experiments (mean ± SD). *P < 0.05; **P < 0.01; ***P < 0.001.

To validate the miRNA expression profiles obtained by deep sequencing, the expression levels of eight randomly selected miRNAs were quantified by qPCR. Figure 3 shows significantly increased expression of two miRNAs (miR-21, let-7i) in the SGC library, and of three miRNAs (miR-142a-3p, miR-223, miR-217) in the RGC library. Thus, the qPCR results were consistent with those obtained by deep sequencing.

### TLR5 is a potential target of cid-miRn-115 regulation

**Figure 4:**
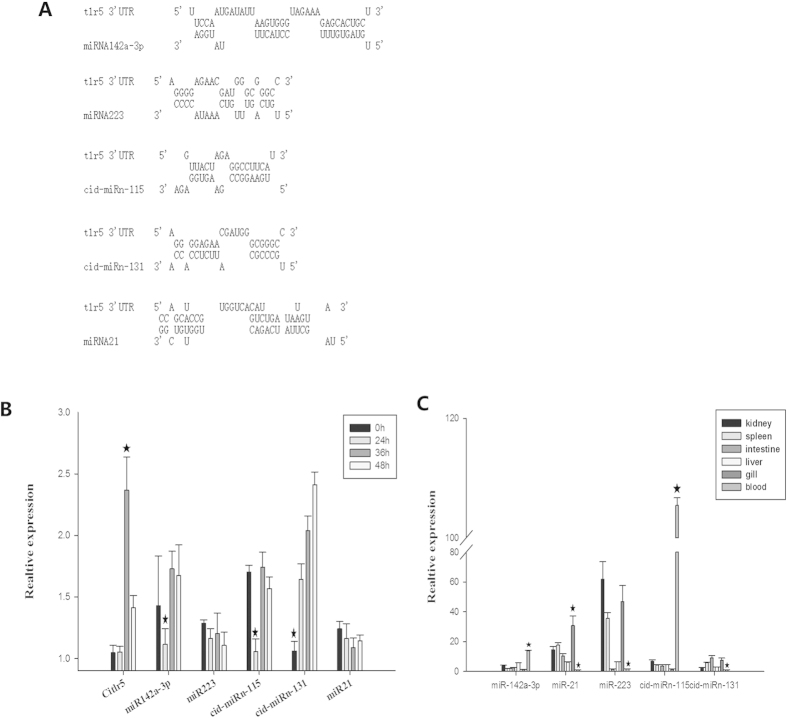
TLR5 is a potential target of miRNA regulation. (**A**) The alignment between miR142a-3p, miR-21, miR-223, cid-miRn-115, cid-miRn-131 and the 3′-UTR segment of *tlr5*. (**B**) CIK was exposed to *FLG22* for 0 h, 24 h, 36 h and 48 h. The expression of *Citlr5* in CIK was detected using real-time PCR. 18S rRNA expression was detected as internal control for mRNA. MiR-192 expression was detected as the internal control for miRNA. (**C**) MiRNA samples were extracted from different tissues, including kidney, spleen, intestine, liver, gill and blood. MiRNA expression was detected by qRT-PCR. MiR-192 was used as loading control. The data were obtained from three independent experiments (mean ± SD). *P < 0.05.

Based on the target scan analysis, we were able to determine miRNA (miR142a-3p, miR-21, miR-223, cid-miRn-115 and cid-miRn-131) binding sites of *Citlr5* 3′-UTR in grass carp (Fig. 4A). Toll-like receptor 5 (TLR5) binding to bacterial flagellin activates NF-κB signalling and triggers an innate immune response to the invading pathogen^21^. Thus, we postulated that the expression of *Citlr5* would be upregulated and TLR5-inhibitory miRNAs would be downregulated upon flagellin challenge. Our results showed that the *Citlr5* expression was time-dependent expression pattern upon flagellin challenge (Fig. 4B), which is consistent with findings from other studies^22^,^23^. Meanwhile, we also observed a inverse expression correlation in cid-miRn-115 and miR-142a-3p with *tlr5* expression. However, we did not detect any expression change for other miRNAs. The inverse expression correlation between cid-miRn-115, miR-142a-3p and *Citlr5* suggests that cid-miRn-115 and miR-142a-3p directly regulate *Citlr5* expression in grass carp.

In addition, we performed a qRT-PCRs experiment to detect the miRNA expression patterns in grass carp. miR142a-3p, miR-21, miR-223, cid-miRn-115 and cid-miRn-131 were found to be expressed ubiquitously in kidney, spleen, intestine, liver, gill and blood (Fig. 4C). Importantly, cid-miRn-115 and miR-142a-3p expression levels in the blood were higher than that in other tissues. Septicaemia is caused by bacterial infection in the blood (bacteraemia) that often occurs with severe infections. Given that the lowest expression of *tlr5* is also found in blood^24^, we therefore investigated whether a regulatory relationship exists between *Citlr5* and cid-miRn-115 and miR-142a-3p.

### Altered expression of immune response genes in cid-miRn-115 and miR-142a-3p overexpressing CIK cells

**Figure 5:**
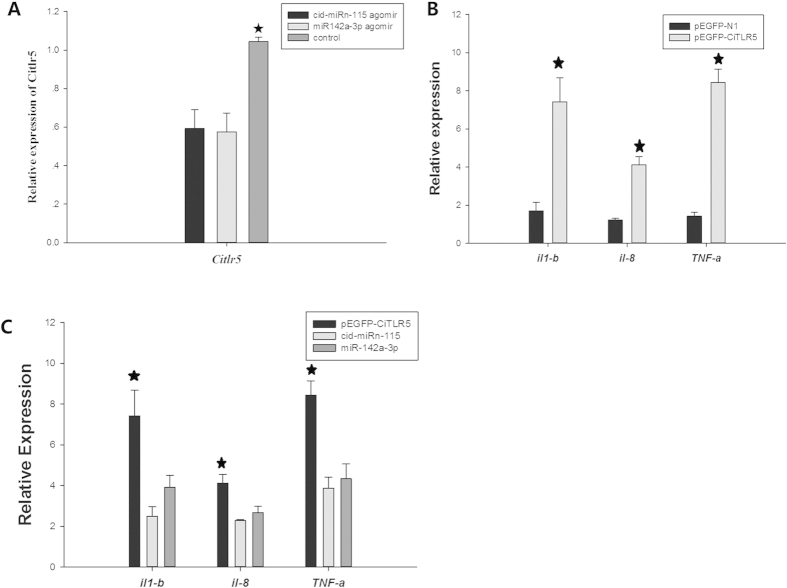
cid-miRn-115 and miR-142a-3p overexpression changes the expression of *Citlr5* and its downstream genes. CIK received cid-miRn-115 and miR-142a-3p agomir at a dose of 50 nM for the indicated times. The relative mRNA expression of *Citlr5* (A) and of TLR5 downstream genes, including *Ciil-1β*, *Ciil-8* and *Citnf-α* (B and C) was detected using real-time PCR. 18S rRNA expression was used as internal control for mRNA. MiR-192 was detected as the loading control. The data were obtained from three independent experiments (mean ± SD). *P < 0.05.

We employed the agomir method to perform a miRNA overexpression of function experiment. We found that in CIK administration of miR-142a-3p and cid-miRn-115 angomir results in a profound up-crease in the endogenous expression of miR-142a-3p and cid-miRn-115 (Fig. S1). Meanwhile, the result showed that the agomir, but not PBS treatment, led to a significant decrease in endogenous *Citlr5* expression (Fig. 5A).

The innate immune response is the first line of defence against infections. The principal challenge for the host is to detect the pathogen and mount a rapid defensive response^25^. TLRs recognise pathogen-associated molecular patterns (PAMPs) and mediate the production of cytokines necessary for the development of effective immunity^26^. After infection with several strains of enteroinvasive bacteria, cells rapidly up-regulate the expression of a program of host genes, the products of which activate inflammatory and immune responses. This inflammatory program includes the upregulated expression and production of proinflammatory and chemoattractant cytokines, such as IL-1β, IL-8 and TNF-α^27^.

Cid-miRn-115 and miR-142a-3p overexpression results in downregulation of *il-1β*, *il-8* and *tnf-α* expression during this process. Indeed, qPCR analysis reveals that cid-miRn-115 and miR-142a-3p overexpression results in a significant decrease in *Citlr5* expression (Fig. 5A), thereby downregulating *Ciil-1β, Ciil-8* and *Citnf-α* expression (Fig. 5B,C).

### Effect of cid-miRn-115 and miR-142a-3p on invasion of A. hydrophila *in vitro*

**Figure 6:**
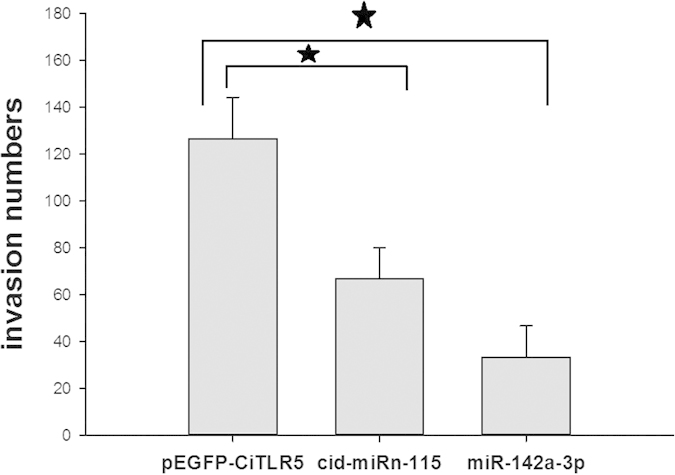
Effect of cid-miRn-115, miR-142a-3p and pEGFP-CiTLR5 on *A. hydrophila* invasion. CIK cells were transfected with plasmid pEGFP- pEGFP-CiTLR5 or cid-miRn-115 and miR-142a-3p agomir. After 24 h, the cells were infected with *A. hydrophila*. The invasion number was counted as the number of entered bacteria. Data are presented as mean ± SE (n = 3). *P < 0.05.

CIK cells were artificially infected with *A. hydrophila* for 45 min after transfection with pEGFP-CiTLR5 or cid-miRn-115 and miR-142a-3p agomir. We observed by microscope (20 × ) that the bacteria successfully infected the cells by insertion. After incubation with gentamicin, free bacteria in the culture medium were killed, which was confirmed by the plate count method. The cells were cleared with Triton X-100 and the numbers of invasive pathogens were also calculated using the plate count method. The number of *A. hydrophila* in cells transfected with cid-miRn-115 and miR-142a-3p was significantly lower than in cells transfected with pEGFP-CiTLR5 (Fig. 6).

## Discussion

miRNAs can affect both the translation and stability of mRNAs^28^. Consistent the notion that miRNAs play a crucial role in controlling gene expression, misregulation of microRNA expression has been found to correlate with several pathologies^29^. In addition to these well-established functions in physiological and pathological processes, it is becoming clear that miRNAs also play crucial roles during microbial infections^1^. To gain further insight into the possible significance of kidney miRNAs in fish, we first identified miRNA expression profiles in the kidney tissue in fish, and then compared miRNAs expression pattern between SGC and RGC kidney samples. We found that nine miRNAs were differentially expressed in the different groups, implying that these differentially expressed miRNAs are involved in bacterial infection. The knowledge of tissue-specific expression pattern of miRNAs can directly inform functional studies^30^. Here, we found that cid-miRn-115, and miR-142a-3p are significantly highly expressed in immune related tissues. Specifically, cid-miRn-115 and miR-142a-3p display a clear time-dependent expression pattern during *FLG22* infection CIK. We thus speculated that cid-miRn-115 and miR-142a-3p might play a key role in regulating the innate immune response.

The innate immune system in fish is considered to be the first line of defence against a broad spectrum of pathogens and is more important in fish than in mammals^31^. The role of miRNAs in innate immune response has been reported for some species, including *Cynoglossus semilaevis*, *Paralichthys olivaceus*, *Cyprinus carpio L.* and *Danio rerio*^32^,^33^,^34^,^35^. In this study, we revealed that cid-miRn-115 and miR-142a-3p are potential regulators of fish innate immune response. Here, we found that cid-miRn-115 and miR-142a-3p are highly expressed in SGC. This study further extends the biological role of cid-miRn-115 and miR-142a-3p in fish.

MiR-142a-3p is a member of the miR-142 family, which has been predicted or experimentally confirmed in a wide range of species. Previous studies have identified miR-142a as a regulator of haematopoiesis, immune system, osteoblast differentiation and fibrosis of the skin by targeting multiple mRNAs^36^,^37^,^38^,^39^. Sequence alignment suggests that the miR-142 family is highly conserved between invertebrates and vertebrates, which indicates that its function might have been conserved. The findings of our study suggest that role of miR-142 in innate immune response is highly conserved between invertebrates and vertebrates.

MiRNAs control biological processes by regulating the expression of their target genes. Here, we found a binding site of cid-miRn-115 and miR-142a-3p in the 3′-UTR region of *Citlr5*, and characterized their effects on *Citlr5* using overexpression assay. Emerging evidence indicates that the TLR5-flagellin interaction plays a central role in driving the inflammatory response triggered by bacteria, and inhibition of *tlr5* normalizes the inflammatory response associated with improved health indicators^40^. In this study, we found that cid-miRn-115 and miR-142a-3p expression stimulate upregulation by *FLG22*, which leads to a gradual decrease in the expression of *Citlr5*.

In that of all known TLRs, only TLR5 can activate proinflammatory gene expression in response to flagellin^41^. A previous study identified TLR5 signalling is associated with increased susceptibility to Legionnaire’s disease^42^. These findings indicate the involvement of TLR5 in inducing *il-8* and *tnf-α*, and in response to pathogenic invasion^24^. Here, we found a similar result, which shows that cid-miRn-115 and miR-142a-3p directly repress *Citlr5* expression. TLR5 directly regulates the expression of multiple genes that are necessary for innate immune response, including *tnf-α*, *il-1* and *il-8* and so on. We also observed an expression change in the downstream gene *Citlr5* when the levels of cid-miRn-115 and miR-142a-3p expression are altered.

Disease outbreaks, some of them caused by pathogenic bacteria, are considered to be one of the largest constraints to development of the aquaculture sector^43^. Similar to terrestrial animal production, antibiotics are also used in aquaculture in an attempt to control bacterial disease^44^. An alternative to killing pathogenic bacteria with antibiotics is to prevent them from attacking the host, without the need to kill them^45^. Quorum sensing pathogens, like the aquatic pathogen *A. hydrophila*, probably increase their chances to infect their host successfully by delaying virulence factor production until the population density is high enough to overwhelm the host immune system^46^. Recognition of microbial pathogens is an essential element for the initiation of innate immune responses. TLR5-deficient mice show increased survival, resulting from decreased migration of bacteria from the intestinal tract to the mesenteric lymph nodes^47^. Thus, TLR5 can be either beneficial or detrimental to the host, depending on the bacterial dose and route of infection^48^. Our results show that *A. hydrophila* in grass carp leads to the activation of *tlr5*. This is similar to a previously reported study in other fish. Cid-miRn-115 and miR-142a-3p overexpression affects the level of *tlr5* expression. We here propose a model for the avoidance of bacterial injury in fish: Once a fish is exposed to a bacterial infection, cid-miRn-115 and miR-142a-3p expression is rapidly upregulated, earlier than other innate immune genes. Cid-miRn-115 and miR-142a-3p upregulation could release *tlr5* inhibition, thus inhibiting its downstream pathway and inflammation reaction. MiRNA-mediated gene regulation operates earlier than most transcriptional responses. The fast regulation of miRNAs after bacterial infection indicates that miRNA-mediated gene silencing acts earlier than most gene transcriptional responses after bacterial damage. Fish have evolved numerous strategies for effectively escaping bacterial injury through distinct signalling pathways. MiRNAs are implicated in buffering developmental processes against the effects of environmental fluctuations.

In summary, we here revealed a novel regulatory mechanism for bacterial infections in fish from miRNA viewpoint. We found that cid-miRn-115 is differentially expressed between SGC and RGC, and its overexpressing using agomir leads to a significant change in *A. hydrophila* invasion and proliferation rates. The post-transcriptional regulation of *tlr5* by cid-miRn-115 and miR-142a-3p could affect the expression of *Citlr5* and its downstream genes, including *il-1β*, *il-8* and *tnf-α*, which in turn affect the immune functions in grass carp. However, samples number and more accurate experimental program should be taken into account in future studies as revealed by miRNA-mediated regulation.

## Additional Information

**How to cite this article**: Xu, X.-Y. *et al.* MicroRNA-induced negative regulation of TLR-5 in grass carp, *Ctenopharyngodon idella*. *Sci. Rep.* **6**, 18595; doi: 10.1038/srep18595 (2016).

## Supplementary Material

Supplementary Table S1

Supplementary Table S2

Supplementary Information

## Figures and Tables

**Table 1:** PCR primer sequences used in this study.

Primer	Sequence (5′–3′)	Application
Let-7a	tgaggtagtaggttgtatagtt	qRT-PCR
Let-7e	tgaggtagtagattgaatagtt	qRT-PCR
Let-7i	tgaggtagtagtttgtgctgtt	qRT-PCR
miR-142a-3p	tgtagtgtttcctactttatgga	qRT-PCR
miR-148	tcagtgcattacagaactttgt	qRT-PCR
miR-21	tagcttatcagactggtgttggc	qRT-PCR
miR-217	tactgcatcaggaactgattgg	qRT-PCR
miR-223	tgtcagtttgtcaaatacccc	qRT-PCR
miR-192	atgacctatgaattgacagcc	qRT-PCR
cid-miRn-115	tgaaggccgaagtggaga	qRT-PCR
cid-miRn-131	tgcccgcattctccacca	qRT-PCR
pEGPF-CiTLR5	F: CCGGAATTCtgATGGGATTTACATTTATTCTGATCC	Overexpression
	R: CGCGGATCCgcTACTGATGTGTTTGCATGGACA	vector construction
*Citlr5*	F: GAAGATAATCTACTTGGGTGAG	qRT-PCR
	R: GTCCGAGATGAAGAAGTTGTAG	
*Ciil-1β*	F: GCCAAGTAGCCGAATCACAGA	qRT-PCR
	R: AGAAGCCCAAGATATGCAGGA	
*Ciil-8*	F: GACGCATTGGTAAACACA	qRT-PCR
	R: TAACCCAGGGAGCAGTAG	
*Citnf-α*	TCACGCTCAACAAGTCTCAG	qRT-PCR
	GAAGTAAATGCCGTCATCAG	
*Ci18s rRNA*	F: GGACACGGAAAGGATTGACAG	qRT-PCR
	R: CGGAGTCTCGTTCGTTATCGG	

**Table 2:** Summary of preliminary analysis of deep sequencing of *A. hydrophila*-susceptible (SGC) and -resistant grass carp (RGC) small RNA libraries.

Category	SGC	RGC
Raw reads	13,284,378	16,095,116
Clean total reads	11,218,000	13,941,337
Total mapped small RNA	3,301,265	4,905,728
Conserved miRNA	61	58
Total conserved miRNA	61	
Novel miRNA	99	108
Total novel miRNA	116	
