# Supplementary material for: MicroRNA-induced negative regulation of TLR-5 in grass carp, Ctenopharyngodon idella
Source: Sci Rep. 2016 Jan 4;6:18595. doi: 10.1038/srep18595 (PMC4698583; doi:10.1038/srep18595)

**MicroRNA-induced negative regulation of TLR-5 in grass carp, *Ctenopharyngodon idella***

Xiao-Yan Xu^a^, Yu-Bang Shen^a^, Jian-Jun Fu^a^, Hong-Yan Yu^a^, Wen-Ji Huang^a^, Li-Qun Lu^c^, Jia-Le Li^a,b*^

^a^Key Laboratory of Exploration and Utilization of Aquatic Genetic Resources, Shanghai Ocean University, Ministry of Education, Shanghai 201306, China;

^b^E-Institute of Shanghai Universities, Shanghai Ocean University, 999 Huchenghuan Road, Shanghai 201306, PR China

^c^National Pathogen Collection Center for Aquatic Animals, College of Fisheries and Life Science, Shanghai Ocean University, 999 Huchenghuan Road, Shanghai 201306, PR China

*Corresponding author: **Jia-Le Li**, Key Laboratory of Exploration and Utilization of Aquatic Genetic Resources, Shanghai Ocean University, Ministry of Education, Shanghai 201306, China

E-mail: [jlli2009@126.com](mailto:jlli2009@126.com);

Phone: +86 021 61900401;

Fax: +86 021 61900405

# Supplementary information

**Table S1**. Conserved miRNAs identiﬁed in *A. hydrophila*-susceptible (SGC) and-resistant grass carp (RGC) small RNA libraries. Sequences: mature sequence of conserved miRNA; SGC(reads): readcount of miRNA in SGC; RGC(reads): readcount of miRNA in RGC; SGC(TPM): the transcripts per million of miRNA in SGC; RGC(TPM): the transcripts per million of miRNA in SGC; Fold change: SGC/RGC; signature: q-value < 0.01 and |log2.Fold change normalized|>1.

**Table S2**. Novel miRNAs predicted in the *A. hydrophila*-susceptible (SGC) and-resistant grass carp (RGC) small RNA libraries. Sequences: mature sequence of novel miRNA; SGC(reads): readcount of miRNA in SGC; RGC(reads): readcount of miRNA in RGC; SGC(TPM): the transcripts per million of miRNA in SGC; RGC(TPM): the transcripts per million of miRNA in SGC; Fold change: SGC/RGC; signature: q-value < 0.01 and |log2.Fold change.normalized|>1.

**Figure legend**

**Figure S1**. Effect of expression angomir treatment on miRNA in CIK cells. CIK cells were received miR-142a-3p or cid-miRn-115 angomir at a dose of 40 nM/well in 6-well plates. The relative expression of miR-142a-3p and cid-miRn-115 was detected using real-time PCR. miR-192 expression was used as the internal control. The data were expressed as the relative change compared with the 0 hour. Asterisk (*) indicates a significant difference compared with the 0 hour (P < 0.05).

**Figure S1**.


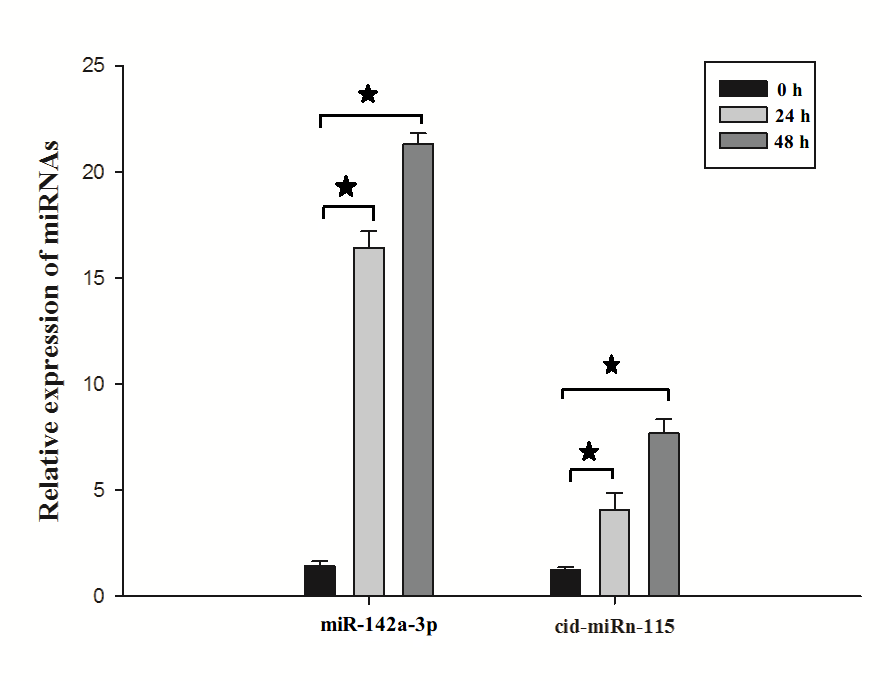

Supplement: Supplementary Information [file srep18595-s3.doc]
